# The Recurrent Mutation in PATL2 Inhibits Its Degradation Thus Causing Female Infertility Characterized by Oocyte Maturation Defect Through Regulation of the Mos-MAPK Pathway

**DOI:** 10.3389/fcell.2021.628649

**Published:** 2021-02-04

**Authors:** Qiqi Cao, Chun Zhao, Congjing Wang, Lingbo Cai, Meng Xia, Xiaolan Zhang, Jian Han, Yangyang Xu, Junqiang Zhang, Xiufeng Ling, Xiang Ma, Ran Huo

**Affiliations:** ^1^State Key Laboratory of Reproductive Medicine, Department of Histology and Embryology, Suzhou Affiliated Hospital of Nanjing Medical University, Suzhou Municipal Hospital, Gusu School, Nanjing Medical University, Nanjing, China; ^2^Department of Reproductive Medicine, Women's Hospital of Nanjing Medical University, Nanjing Maternity and Child Health Care Hospital, Nanjing, China; ^3^Clinical Center of Reproductive Medicine, State Key Laboratory of Reproductive Medicine, First Affiliated Hospital, Nanjing Medical University, Nanjing, China; ^4^Center for Global Health, School of Public Health, Nanjing Medical University, Nanjing, China

**Keywords:** meiotic maturation, PATL2 mutation, ART, MAPK, female infertility

## Abstract

PAT1 homolog 2 (PATL2), encoding an RNA-binding protein, is a repressor involved in the translational regulation of maternal mRNAs during oocyte maturation. Previous studies have reported mutations in *PATL2* those led to female infertility with oocyte maturation arrest; however, the mechanisms by which mutations affected meiotic maturation remained unclear. Here, we identified several novel and recurrent mutations of *PATL2* in patients with similar phenotype, and chose the missense mutation c.649 T>A p.Tyr217Asn in *PATL2* (PATL2^Y217N^) as a typical to investigate the underlying mechanisms. We confirmed that this mutation disturbed oocyte maturation and observed morphological defects of large polar body, symmetrical division and abnormal spindle after microinjection of corresponding mutated mRNA. We further evaluated the effect of the PATL2^Y217N^ mutation in 293T cells, and found this mutation decreased the ubiquitination level and degradation of PATL2. Then, abnormally increased PATL2 bound mRNAs of Mos, an upstream activator of mitogen activated protein kinase (MAPK), to regulate its translational activity and subsequently impaired MAPK signaling pathway and oocyte meiosis. These results dissented from the previous view that *PATL2* mutations reduced their expression and highlight the role of PATL2 in translational regulation of Mos and its association with MAPK signaling pathway during oocyte meiotic maturation.

## Introduction

The wide application of assisted reproductive technology (ART) allows us to access the maturity and classify phenotypes based on the morphology of oocytes upon retrieval (Ebner et al., 2003; Muasher et al., 2006; Beall et al., 2010). In clinical ART, these morphologic appearances are used to generally identify different phases of meiotic progression such as GV (germinal vesicle), MI (metaphase I), or MII (metaphase II) oocyte. Only when the oocyte undergoes the meiotic division into MII, it can be successfully fertilized. Therefore, the meiotic maturation of oocytes is an integral part of reproduction. Meiotic progression depends upon the precise control of a large number of mRNAs and proteins synthesized in growing oocytes (Huo et al., 2004; Liang et al., 2007). It is accomplished through the regulation of protein translation, phosphorylation, and degradation (Sorensen and Wassarman, 1976; Rauh et al., 2005). The most common pathways for degradation of maternal proteins are autophagy lysosome pathway and the ubiquitin proteasome pathway (Dikic, 2017; Varshavsky, 2017). For example, cytoplasmic polyadenylation element binding protein 1 (CPEB1) is a key regulator for maternal mRNA translation but undergoes ubiquitin-dependent degradation during meiosis I otherwise it can affect the progression to meiosis II (Setoyama et al., 2007; Ivshina et al., 2014). In this respect, protein degradation may be viewed as a prerequisite to complete the oocyte maturation and achieve oocyte-to-zygote transition. Any subtle defect in these procedures could result in maturation failure.

Knockout mouse models were previously used to discover many key factors of oocyte maturation (Holt et al., 2013), but there was still limited evidence of their pathogenicity to human reproduction. Recently, several genes were reported to be responsible for human oocyte maturation arrest. *TUBB8* mutations and *TRIP13* mutations were identified to cause human oocyte MI arrest (Feng et al., 2016; Zhang et al., 2020). With increasing attention, other variable phenotypes of the TUBB8 mutations have also been discovered (Chen et al., 2017a; Huang et al., 2017; Wang et al., 2018; Xiang et al., 2018; Yuan et al., 2018; Jia et al., 2020; Xing et al., 2020). The same as *TUBB8* gene, pathogenic variants in *PATL2* was first found to result in GV arrest (Chen et al., 2017b; Maddirevula et al., 2017), and then similar but slightly variable phenotypes were reported (Chen et al., 2017b; Maddirevula et al., 2017; Christou-Kent et al., 2018; Huang et al., 2018; Wu et al., 2019; Liu et al., 2020). Although the affected cases of *PATL2* mutations are accumulating, the molecular etiology underlying this remains largely unknown.

In this study, we identified seven mutations within PATL2 by whole-exome sequencing (WES), including three recurrent mutations (c.898C>T p.Gln300^*^, c.1376C>A p.Ser459Tyr, c.649T>A p.Tyr217Asn) and four novel mutations (c.1345A>G p.Thr449Ala, c.931A>G p.Met311Val, c.920G>A p.Arg307Gln, c.1336C>T p.Arg446Trp) in individuals affected with primary infertility. Considering the conservation of mutations among different species and the consistency of phenotype among patients, we selected the recurrent mutation p.Tyr217Asn in *PATL2* to confirm its pathogenicity in mouse oocytes, then further investigated the effect of the missense mutation and attempted to rescue the impaired phenotype by *in vitro* studies. Our findings show a novel perspective that mutation of *PATL2* caused the abnormal accumulation of its protein, which is different from previous studies and might provide a prospect of improved IVM protocol for future therapies for affected individuals.

## Materials and Methods

### Human Subjects and Ethics Approval

Infertility patients were recruited from the Reproductive Medicine Center of Women's Hospital of Nanjing Medical University and The First Affiliated Hospital of Nanjing Medical University. All samples from donors were obtained with informed consent. This study was approved by the Ethics Committee of the Nanjing Medical University (2018/651), the Ethics Committee of Women's Hospital of Nanjing Medical University (KY-025) and the Ethics Committee of The First Affiliated Hospital of Nanjing Medical University (2012-SR-128).

### Genomic DNA Extraction and Genetic Analysis

Genomic DNA samples were extracted from peripheral blood with a RelaxGene Blood DNA System using standard methods (Tiangen, Beijing, China, DP319). Whole-exome sequencing was performed to identify candidate variants. Whole-exome capture used the Aglient SureSelect Human All Exon V6 (Agilent), and sequencing was carried out on the Illumina NovaSeq 6000 platform (Illumina) by Microanaly Genetech Co., Ltd. (Anhui). We selected candidate variants with the following criteria: (1) had a under 1% frequency in public databases (such as the genome Aggregation Database (gnomAD) and the Exome Aggregation Consortium (ExAC) Browser), (2) variants located in exon or splice site, (3) filtration using our in-house gene list that were highly expressed or specifically expressed in oocytes or early embryo, (3) sanger sequencing: PCR amplifications were performed with corresponding primers using 2X Rapid Taq Master Mix (Vazyme, Nanjing, P222) to validate the candidate variant in the affected individuals and their parents if available (Primers in Supplementary Table 1). Subsequently, the conservation analysis among multiple species was performed using MEGA software. And the functional effect of mutations was assessed by Sorting Intolerant from Tolerant (SIFT,) and Polymorphism Phenotyping (Polyphen2).

### Vector Construction and *In vitro* Transcription

Wild-type human PATL2 were constructed and then recombined with the eukaryotic expression vector pcDNA3.1. A MYC-tag or a FLAG-tag were fused at N-terminus of PATL2. The vectors were constructed by GenScript (Nanjing). The variant c.649T>A was generated using Fast Mutagenesis Kit (Vazyme, Nanjing, C214). GFP reporter plasmid for analyses of Mos 3′-UTR activities were constructed by GenScript (Nanjing) (Dai et al., 2019). The PATL2^WT^-MYC and PATL2^Y217N^-MYC plasmids were linearized with XbaI enzyme (New England Biolabs, #R0145V) and then be transcribed to PATL2^WT^ and PATL2^Y27N^ cRNAs using HiScribe T7 ARCA mRNA Kit (New England Biolabs, E2060) according to the manufacturer's standard mRNA synthesis protocols. Mos 3′-UTR reporter plasmid was linearized and *in vitro*-transcribed using the SP6 mMESSAGE mMACHINE Kit (Invitrogen, AM1340) according to the manufacturer's instructions.

### Cell Culture and Transfection

HEK293T cells were maintained in Dulbecco's modified Eagle's medium (DMEM, Life Technologies/Gibco, Grand Island, New York, #11995073) supplemented with 10% fetal bovine serum (FBS) (Life Technologies/Gibco, Grand Island, NY, #10270106), 10,000 units/ml of penicillin and 10,000 μg/ml of streptomycin (Invitrogen, 15140-122), and the cells were cultured at 37°C with 5% CO_2_. Cells were transiently transfected for 6 h using Lipofectamine 2000 reagent (Invitrogen, USA, #11668019), then cells were washed twice with PBS and maintained in serum-free medium for 48 h before harvesting. Twenty-four hours post transfection with the PATL2^WT^-FLAG or PATL2^Y217N^-FLAG plasmids, the HEK293T cells were treated with proteasome inhibitor MG132 (Merck, M7449) at a concentration of 10 μM for 6 h and analyzed by western blotting. At 24 h post-transfection, cells were treated with CHX cycloheximide (CHX) (APEXBIO, A8244), an inhibitor of new protein synthesis, at a concentration of 10 μM for the indicated amount of time and harvested periodically and analyzed by western blotting.

### Oocyte Collection and Microinjection

Female ICR mice (4 week) were used for oocyte collection. To collect fully grown GV oocytes, mice were superovulated by intraperitoneal injection with 5 IU pregnant mare serum gonadotropin (PMSG) (Ningbo Sansheng Pharmaceutical Corporation, Zhejiang, China). Cumulus-enclosed oocytes were obtained by manual rupturing of antral ovarian follicles 48 h later. To obtain fully grown GV oocytes, cumulus cells were removed by repeatedly pipetting. For *in vitro* maturation, GV oocytes were cultured in M16 medium (Sigma, M7292) under mineral oil (Sigma, M8410) at 37°C in a 5% CO_2_ incubator. For microinjection, fully grown GV oocytes were harvested in M2 medium (Sigma, M7167) with 2.5 μM milrinone (Sigma, M4659) to inhibit meiotic resumption. Approximately 10 pL of complementary RNA was injected at a concentration of 1,000 ng/μl. After injections, oocytes were arrested at the GV stage in M2 medium containing 2.5 μM milrinone for 12 h to allow sufficient translation, then washed in milrinone-free M16 medium, and cultured for 3 h to observe meiotic resumption (GVBD) or 14 h to detect the first polar body (Pb1) extrusion. We collected the oocytes after milrinone removal for further analysis.

### Western Blots

Cell protein concentrations were determined with a BCA Protein Assay (Beyotime Biotechnology, China, P0012). Oocyte protein concentrations were adjusted by the equal number of oocytes. The protein was separated by 10% sodium dodecyl sulfate-polyacrylamide gel electrophoresis before being transferred to polyvinylidene fluoride membranes (Millipore, Massachusetts, IPVH00010). Non-specific binding sites were blocked for 2 h at room temperature with 5% non-fat milk in Tris-buffered saline containing 0.05% Tween-20. Membranes were incubated overnight at 4°C with a dilution of the following antibodies: GAPDH (Abclonal, China, AC002), ACTIN (Proteintech, China, #66305-1-lg), β-TUBULIN(Abclonal, AC021), MYC (Cell Signaling Technology, Massachusetts, #2278), FLAG (SIGMA, USA, F7425), Ubiquitin(Santa Cruz, sc-8017), PATL2 (Invitrogen, PA5-48187), ERK1(Santa Cruz, sc-271269) and pERK1/2(Cell Signaling Technology, 9101s). After incubation with an anti-immunoglobin horseradish peroxidase-linked antibody (Invitrogen, USA, #31430 and #31460) for 1 h, the immune complexes were detected by enhanced chemiluminescence (FDBIO, China, FD8020). For densitometric analyses, protein bands on the blots were measured by ImageJ software.

### Coimmunoprecipitation (Co-IP) Assays

Cells were harvested and lysed with RIPA lysis buffer (Beyotime, P0013K) containing 1 mM protease inhibitor cocktail (Bimake, B14001) on ice for 40 min. Then, the cells were centrifuged at 12,000 rpm for 30 min at 4°C. Protein lysates were incubated with 80 μL of Protein A/G Magnetic Beads (Bimake, Shanghai, B23202) after preclearing the beads for 1 h. Then, 8 μg of anti-FLAG antibody (SIGMA, F7425) were added, and mix was subjected to gentle rotation overnight at 4°C. Western blots were operated after the beads were washed six times with lysis buffer.

### Immunofluorescence and Confocal Microscopy

Oocytes were fixed in phosphate-buffered saline (PBS) supplemented with 4% paraformaldehyde (PFA) (Sigma, P6148) for 30 min at room temperature and then incubated in 0.5% Triton X-100 for 30min at 37°C. Then, samples were blocked in 1% bovine serum albumin in PBS for 1 h and incubated with primary antibodies overnight at 4°C. After washed three times in PBS, the samples were incubated with secondary antibodies for 1 h at room temperature. After washed three times in PBS, the samples were stained with Hoechst 33342 (KeyGen BioTECH, KGA212-10) for 10 min and observed by confocal microscopy (LSM 800; Carl Zeiss, Germany).

### Statistical Analyses

Statistical differences between groups were conducted by Student's *t*-test when appropriate. Derived values are presented as the means ± SD. *p* < 0.05 were considered statistically significant. “n.s.” refers to non-significant.

## Results

### Clinical Characterization

**Figure 1:**
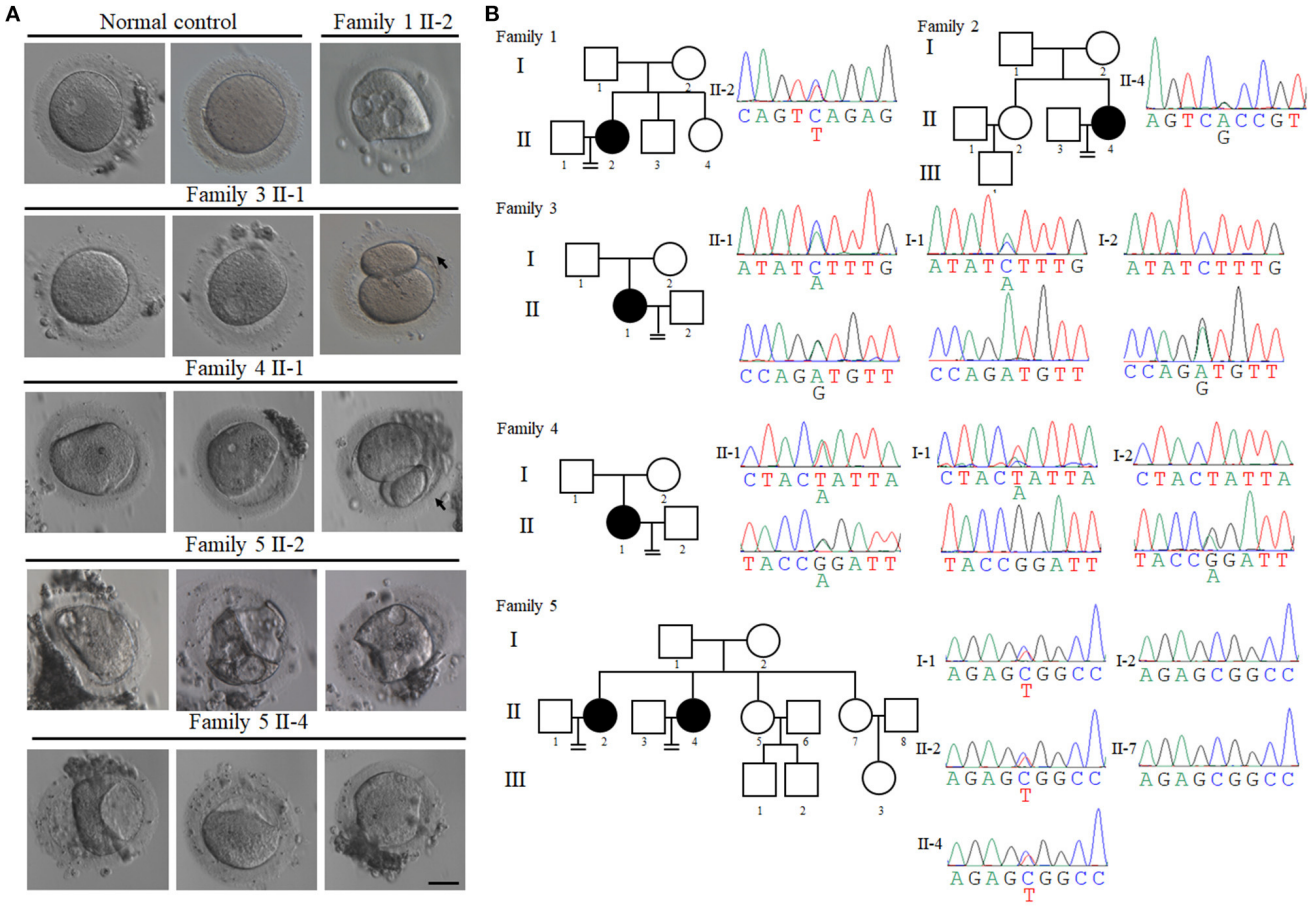
Clinical characterization and identification of *PATL2* mutations in the infertile patients. **(A)** The morphology of oocytes [germinal vesicle (GV), metaphase I (MI), and first polar body (Pb1) oocytes] from normal and affected individuals with PATL2 mutations. Individuals from family 3 and 4 had Pb1 oocytes with abnormal polar body, as indicated by the arrows. The scale bar represents 50 μm. **(B)** Pedigrees of five affected families. Sanger sequencing confirmation is shown below the pedigrees. Solid indicates affected members, and equal signs denote infertility. Sanger sequencing result is shown beside the pedigrees. The mutations in the affected individuals from families 3, 4, and 5 were inherited from their parents. The patients in families 1 and 2 had unknown inheritance patterns. The patients in families 1 carried mutation c.C898T p.Q300* and family 2 carried mutation c.A1345G p.T449A. The patients in families 3 had the compound heterozygous mutations c.C1376A p.S459Y and c.A931G p.M311V. Compound heterozygous mutations c.G920A p.R307Q and c.T649A p.Y217N were identified in family 4. Heterozygous mutation c.C1336T p.R446W was identified in two sisters in family 5.

The first patient (family 1 II-2) had not conceived after 3 years of attempts without contraception, and she was diagnosed with primary infertility at the age of 25. She had regular menstrual cycles and normal sex hormone concentrations (Table 1). She underwent three *in vitro* fertilization (IVF) attempts. A total of 33 oocytes were retrieved, of which 17 GV oocytes were retrieved and the remaining 16 oocytes degenerated (Figure 1A and Table 1). The second patient (family 2 II-4) was 28 years old and had been diagnosed with infertility 3 years earlier. In her first IVF treatment attempt, four MI oocytes and one matured Pb1 oocyte were retrieved. Only one degenerated oocyte was retrieved in her second IVF treatment cycle (Table 1). The third patient (family 3 II-1) was 28 years old and had been diagnosed with primary infertility of unknown causes for 7 years. Thirty oocytes were retrieved in two IVF cycles, including 17 GV oocytes, five MI oocytes, four degenerated oocytes, three Pb1 oocyte with abnormal morphology, and one fertilized oocyte but arrested at early stage (Figure 1A and Table 1). The forth patient (family 4 II-1) was 32 years old and during her 4 years of infertility, she underwent one IVF cycle. She had a similar phenotype that two Pb1 oocytes with large polar body were retrieved apart from four immature GV oocytes (Figure 1A and Table 1). Two sisters from family 5 (family 5 II-2 and II-4) had been diagnosed with primary infertility for several years, although they all had regular menstrual cycles and normal sex hormone concentrations. They had same phenotypes in their IVF treatments, such that all immature oocytes were arrested at GV stage (Figure 1A and Table 1).

**Table 1:** Clinical features of the patients.

**Patient**	**Age**	**Duration of Infertility (Years)**	**BMI**	**Basal sexual hormone**					**Cycle**						
				**FSH^**a**^ (IU/L)**	**LH^**b**^ (IU/L)**	**E^**c**^ (pg/ml)**	**Testo (ng/dl)**	**AMH (ng/ml)**	**IVF/ICSI Cycles**	**Total Number of Oocytes Retrieved**	**GV Oocytes**	**MI Oocytes**	**Pb1 Oocytes**	**Fertilized Oocytes**	**Degenerated oocytes**
Family1 II-2	25	3	20.2	7.86	5.62	52	–	7.92	1(IVF)	27	13	0	0	0	14
									2(IVF)	1	0	0	0	0	1
									3(IVF)	5	4	0	0	0	1
Family2 II-4	28	3	19.3	5.45	3.29	81.47	1.15	2.56	1(IVF)	5	0	4	1	0	0
									2(IVF)	1	0	0	0	0	1
Family3 II-1	28	7	20	6.9	7.74	270.7	0.93	5.9	1(IVF)	15	8	1	3	1	2
									2(IVF)	15	9	4	0	0	2
Family4 II-1	32	4	20.4	7.9	5.13	42	0.41	8.15	1(IVF)	6	0	4	2	0	0
Family5 II-2	31	7	18.4	10.33	4.04	29	0.67	1.09	1(IVF)	12	12	0	0	0	0
Family5 II-4	28	7	21.5	8.76	4.64	76	0.33	3.54	1(IVF)	7	7	0	0	0	0

### Identification of Mutations in *PATL2*

**Figure 2:**
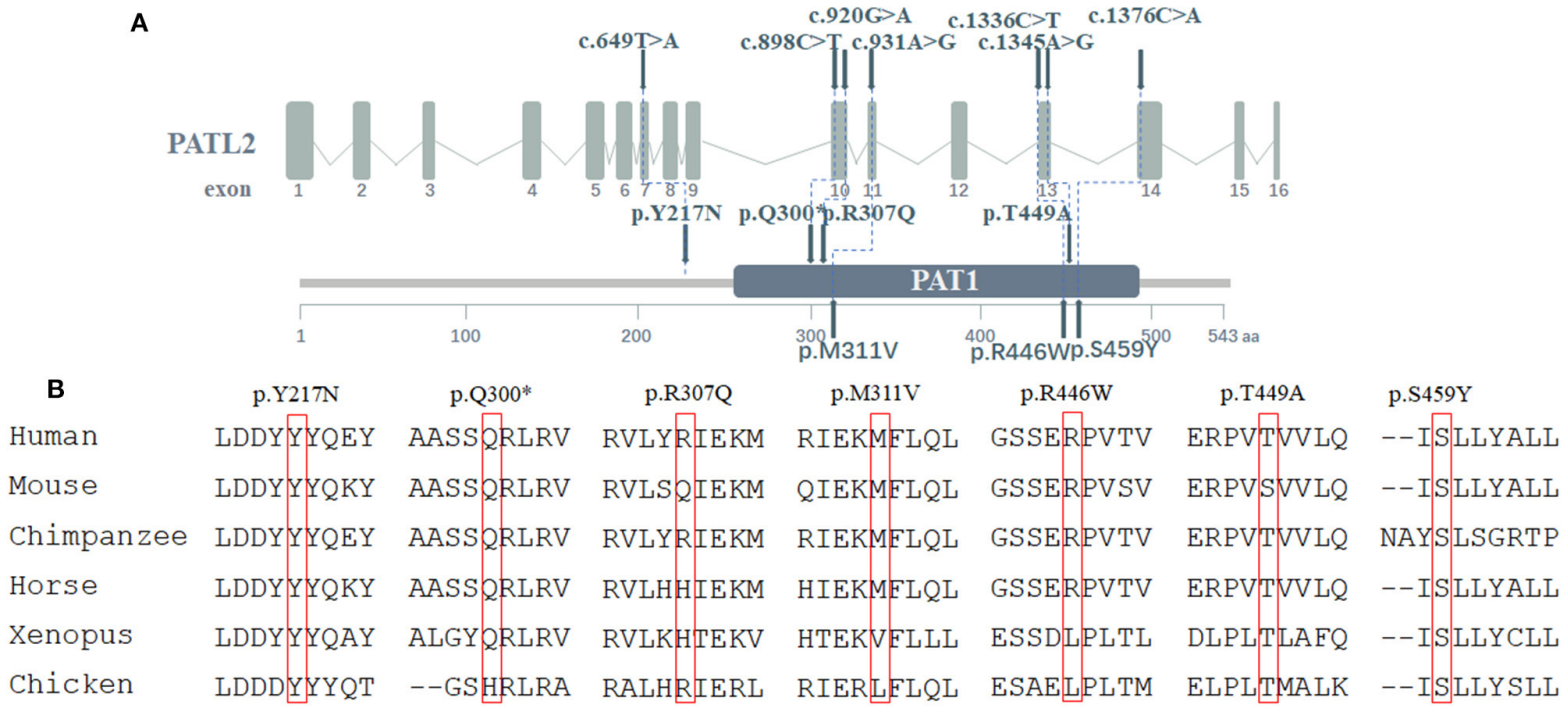
Location and conservation of mutations in *PATL2*. **(A)** The positions of *PATL2* mutations and functional domains are indicated in the gene structures. **(B)** Conservation of mutated sites in PATL2 among species.

To investigate the genetic cause of the infertility, we performed WES of affected individuals. After bioinformatics analysis based on our filtering protocols (see section Materials and Methods), we identified mutations in *PATL2* which may be responsible for the phenotypes of these infertile females. We first identified a likely pathogenic heterozygous non-sense mutation c.898C>T (p.Gln300^*^) of *PATL2* (NM_001145112.1) in the patient (family 1 II-2) and the mutation was verified by sanger sequencing (Figures 1B, 2A, Table 2). A heterozygous missense variant in *PATL2* c.1345A>G (p.Thr449Ala) was identified in the patient (family 2 II-4). Subsequent sanger sequencing was performed to confirm the variant site which may be involved in the maturation-defective phenotype (Figures 1B, 2A, Table 2). We also found two other families (family 3 and 4) carrying *PATL2* compound heterozygous pathogenic variants. The proband in family 3 had the compound heterozygous variants c.1376C>A (p.Ser459Tyr) and c.931A>G (p.Met311Val) of *PATL2*. The affected individual in family 4 carried the compound heterozygous variants c.920G>A (p.Arg307Gln) and c.649T>A (p.Tyr217Asn). Sanger sequencing was then used to validate these mutations, and it was confirmed that the variants derived from both their parents (Figures 1B, 2A, Table 2). Another heterozygous missense variant in c.1336C>T (p. Arg446Trp) in exon 13 of *PATL2* was identified in two infertile sisters (family 5 II-2 and II-4) of family 5. Subsequently, sanger sequencing showed that the affected patients (family 5 II-2 and II-4) and their father (family 5 I-1) carried the heterozygous *PATL2* mutation, while her mother (family 5 I-2) and their fertile sister (family 5 II-7) did not, indicating a dominant pattern of inheritance (Figures 1B, 2A, Table 2). The altered amino acid substitutions except mutation Y217N were all located in the conserved PAT1 domain of PATL2 (Figure 2A). The functional impact of all variants was assessed by SIFT and PolyPhen 2 (Table 2). Moreover, the positions where these mutations in *PATL2* occur are highly conserved among most species (Figure 2B).

**Table 2:** Overview of *PATL2* mutations identified in the patients.

**Patient**	**Mutation gene**	**Genomic position (bp)**	**cDNA change**	**Protein change**	**Mutation type**	**Genotype**	**SIFT**	**Polyphen2**	**ExAC_EAS**	**gnomAD_EAS**
Family1 II-2	PATL2	Chr15: 44961740	c.C898T	p.Q300^*^	Stopgain	Heterozygous	NA	NA	NA	NA
Family2 II-4		Chr15: 44960560	c.A1345G	p.T449A	Missense Mutation	Heterozygous	Tolerated	Benign	NA	NA
Family3 II-1		Chr15: 44959391	c.C1376A	p.S459Y	Missense Mutation	Heterozygous	Damaging	Damaging	NA	NA
		Chr15: 44961611	c.A931G	p.M311V	Missense Mutation	Heterozygous	Tolerated	Benign	NA	NA
Family4 II-1		Chr15: 44961718	c.G920A	p.R307Q	Missense Mutation	Heterozygous	Tolerated	Benign	NA	NA
		Chr15: 44964221	c.T649A	p.Y217N	Missense Mutation	Heterozygous	Damaging	Damaging	NA	NA
Family5 II-2/4		Chr15: 44960569	c.C1336T	p.R446W	Missense Mutation	Heterozygous	Damaging	Damaging	NA	NA

* *Means the mutant sequence corresponds to stop codon sequence*.

### Confirmation the Pathogenicity of Y217N Variant in Mouse Oocyte

**Figure 3:**
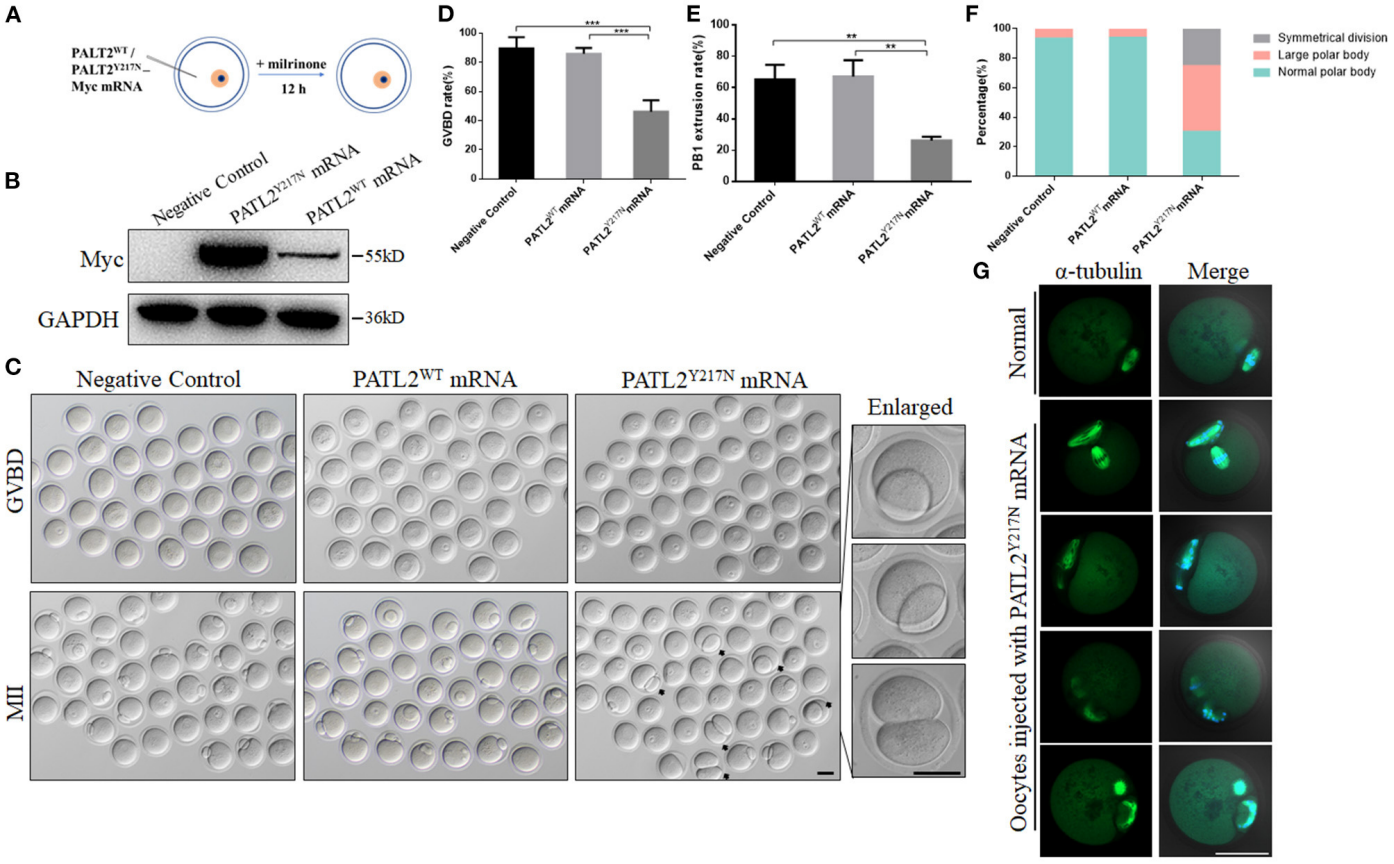
Pathogenicity of Y217N variant in mouse oocyte. **(A)** Illustration of mRNA microinjection in **(B–G)**. **(B)** Expression of PATL2^WT^ and PATL2^Y217N^ cRNA after microinjection was verified by Western blot analysis. **(C)** Phase-contrast images of negative control and PATL2^WT^ or PATL2^Y217N^ cRNA injected oocytes. Black arrows indicate oocytes with apparent abnormal polar body. Enlarged images of oocytes with large polar body and symmetrical division are arranged in the right line. Scale bars indicate 50 μm. **(D,E)** Quantitative analysis of GVBD rate **(D)** and Pb1 extrusion rate **(E)** Graphs show means ± SD of results observed in three independent experiments. **P < 0.01, ***p < 0.001 **(F)** The proportion of the normal polar body, large polar body and symmetrical division in negative control and PATL2^WT^ or PATL2^Y217N^ cRNA injected oocytes. Total number of oocytes analyzed: *n* = 184 for negative control oocytes; *n* = 200 for PATL2^WT^ cRNA injected oocytes; *n* = 74 for PATL2^Y217N^ cRNA injected oocytes. *P* < 0.017 was considered to be statistically significant by Pearson chi square test. **(G)** Spindle defects and chromosome misalignment were detected in PATL2^Y217N^ cRNA injected oocytes. Scale bar indicates 50 μm.

To investigate the effect of PATL2 mutation during oocyte meiotic maturation, we first constructed wild-type PATL2, mutant PATL2 (p.Tyr217Asn) and then recombined with the eukaryotic expression vector pcDNA3.1 with a MYC-tag. The wild-type PATL2 and mutant PATL2 plasmids were transcribed into complementary RNAs (cRNA) *in vitro* and microinjected into mouse GV oocytes (Figure 3A). The expressions of cRNA were determined by western blot (Figure 3B). After 3 h culture for *in vitro* maturation, both negative control and PATL2^WT^ groups resume meiosis normally, indicated by the similar GVBD rate over 80%. However, the GVBD rate largely decreased in PATL2^Y217N^ group (Figures 3C,D). Moreover, only 23.4% PATL2^Y217N^ cRNA-injected oocytes extruded Pb1 after 14 h culture, which was significantly reduced compared to PATL2^WT^ cRNA-injected and negative control oocytes (Figures 3C,E). It is worth noting that large polar body and 2-cell like symmetrical division were observed in a number of oocytes which appeared to have been completed the meiotic maturation (Figure 3C, Enlarged and Figure 3F). In addition, critical events during oocyte maturation process such as spindle assembly and chromosome alignment were observed in PATL2^Y217N^ cRNA-injected oocytes by immunofluorescent staining. We found that the Y217N mutation caused spindle disorganization and chromosome misalignment in oocyte meiosis (Figure 3G). Together, these results suggest that PATL2 is essential for orderly meiosis during oocyte maturation and the p.Tyr217Asn mutation of PATL2 impairs the progress.

### Effects of Y217N Variant on PATL2 Degradation

**Figure 4:**
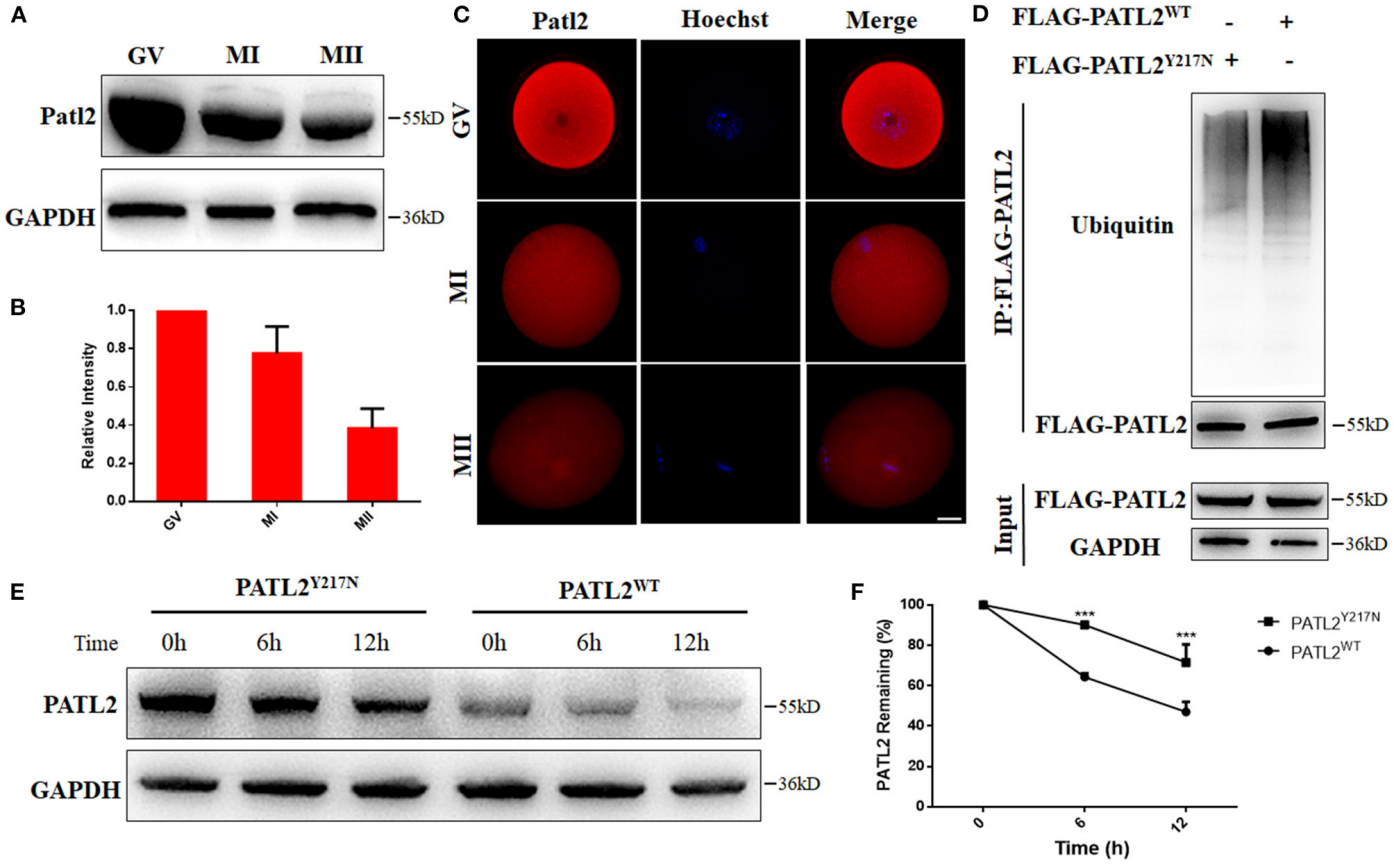
Effects of Y217N variants on PATL2 degradation. **(A,B)** The Patl2 levels in oocytes at germinal vesicle (GV), meiosis metaphase I (MI), and meiosis metaphase II (MII) stages were determined by western blot. **(C)** Immunostaining of Patl2 in oocytes at GV, MI, and MII stages. Scale bar, 20 μm. **(D)** Ubiquitination was evaluated by co-IP with an anti-FLAG antibody. **(E,F)** The PATL2 stability comparison between group of PATL2^WT^ or PATL2^Y217N^ transfected cells is followed by CHX treatment. ***p < 0.001.

To investigate the importance of Patl2 during oocyte meiotic maturation, we first performed western blot analysis and immunofluorescence to characterize the expression pattern of Patl2 in mouse oocytes. As shown in Figures 4A,B, Patl2 level was abundant in GV oocytes and decreased during meiotic maturation. Furthermore, subcellular localization of Patl2 was distributed in the cytoplasm with lighter staining during meiotic maturation (Figure 4C). These results indicate that PATL2 might play a pivotal role during the process of oocyte maturation. It has been previously reported that Xenopus Pat1a, the PATL2 ortholog, degraded by proteolysis via PEST-like region (Rechsteiner and Rogers, 1996; Marnef et al., 2010). In addition, we used an integrated bioinformatics platform to predict that PATL2 has multiple E3 recognizing motifs, one of which is adjacent to Tyr217 residue (http://ubibrowser.ncpsb.org) (Li et al., 2017). Therefore, we asked whether the p.Tyr217Asn mutation influenced the ubiquitin-mediated degradation of PATL2 protein. To answer this question, we transfected FLAG-PATL2^WT^ or FLAG-PATL2^Y217N^ into 293T cells with proteasome inhibitor MG132 treatment to determine the influence of the ubiquitination level *in vitro*. Immunoprecipitation analysis showed decreased ubiquitination level in the groups transfected with mutant PATL2 pulled down by anti-Flag beads (Figure 4D). We next sought to analyze the effect of the PATL2^Y217N^ on degradation of PATL2. While the protein level of PATL2^WT^ was decreased progressively after 6 h and 12 h after treatment with CHX, an inhibitor of new protein synthesis, the protein level of PATL2^Y217N^ was relatively stable (Figures 4E,F). These data indicated that the mutation affected its ubiquitination, and caused the abnormal accumulation of PATL2.

### Regulation of MOS Translational Activity and MAPK Signaling Pathway by Increased PATL2 Level

**Figure 5:**
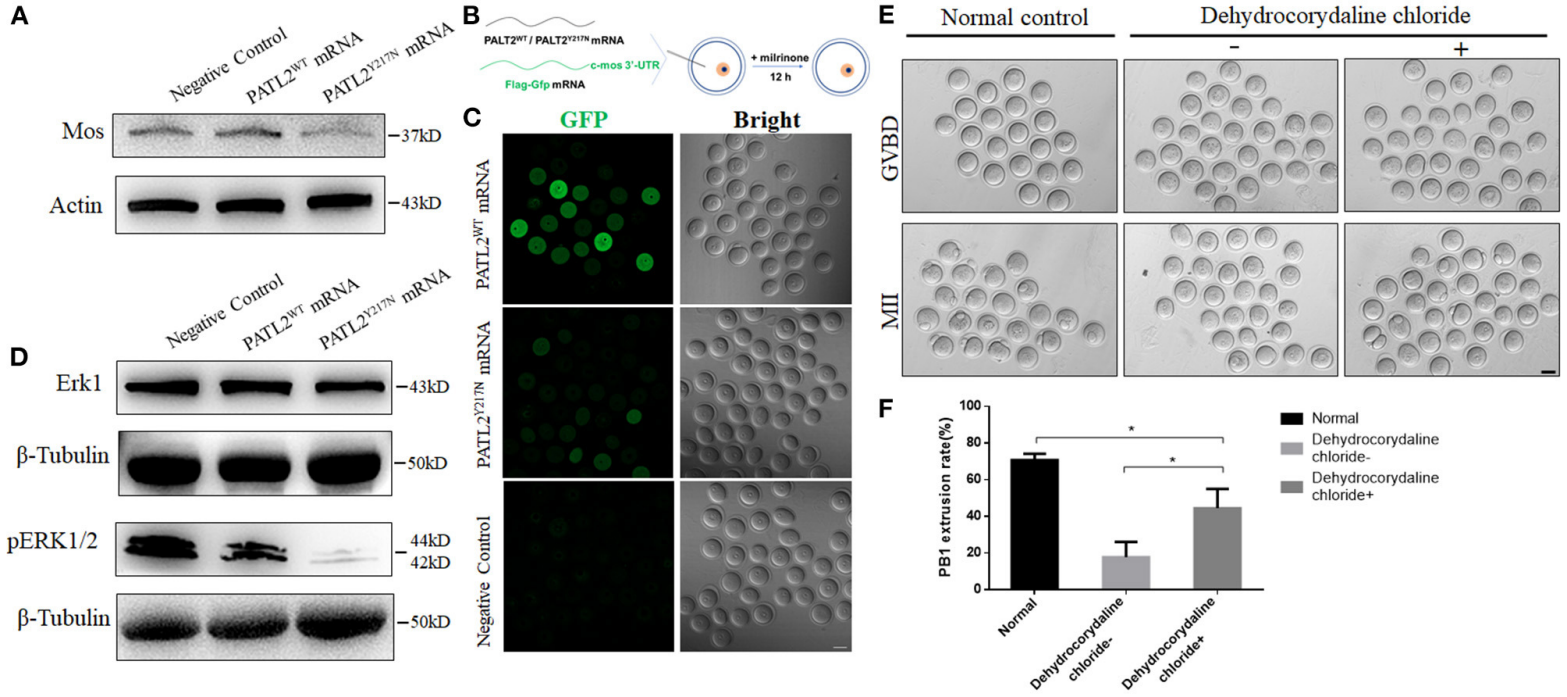
Repression of MOS with its cascade effect and phenotypic rescue by dehydrocorydaline chloride. **(A)** The MOS level in oocyte groups of negative control, PATL2^WT^ or PATL2^Y217N^ cRNA microinjected were determined by western blot. **(B)** Illustration of mRNA microinjection and oocyte culture. **(C)** Fluorescence results showing the expression of GFP fused with Mos 3′-UTR. The scale bar represents 50 μm. **(D)** ERK1and pERK1/2 protein levels in in oocytes of negative control, PATL2^WT^ or PATL2^Y217N^ cRNA microinjected were determined by western blot. **(E)** Representative images of normal control oocytes and that of PATL2^Y217N^ cRNA injected oocytes were cultured with/without dehydrocorydaline chloride. Scale bars indicate 50 μm. **(F)** Quantitative analysis of Pb1 extrusion rate. Data are represented as the mean ± SD; *p < 0.05.

In Xenopus, overexpression of P100 (the ortholog of human PATL2) not only represses translation, but also functions to inhibit oocyte maturation by affecting c-mos accumulation (Nakamura et al., 2010). To explore the defect of abnormally increased PATL2, we performed western blot analysis to detect the expression of MOS, and found an obvious reduction in oocytes microinjected PATL2^Y217N^ mutated RNA (Figure 5A). To further investigate the mechanism of MOS reduction, we constructed eGFP reporter fused with the 3′-untranslated region (3′-UTR) of c-mos and transcribed it *in vitro*, then mixed it with PATL2^WT^ or PATL2^Y217N^ RNA, and microinjected them into GV oocytes (Figure 5B). The oocytes were arrested in M2 medium supplemented with 2.5 μM milrinone for 12 h. Subsequently, we performed immunofluorescence assay and the fluorescence intensity was analyzed. We detected weak GFP signals in PATL2^Y217N^ group, whereas that in PATL2^WT^ group was strong (Figure 5C). In oocytes, Mos, a specific upstream regulator of MAPK, plays a crucial role in controlling meiotic maturation (Dupre et al., 2011). As expected, phosphorylated ERK1/2 (pERK1/2), which was well-known as a marker of MAPK pathway activation reduced in MII oocytes of PATL2^Y217N^ group (Figure 5D). Next, we doubt whether activated MAPK pathway could rescue the pathogenic effects. The MAP kinase was activated 2 h after GVBD (Sun et al., 1999). Thus, PATL2^WT^ or PATL2^Y217N^ cRNA injected oocytes were cultured *in vitro* for 4 h to GVBD stage then transferred into M16 medium with 2 μM dehydrocorydaline chloride for 1 h, which could elevate p38 MAPK activation. Subsequently, the oocytes continued culturing *in vitro* for the analysis after washed thoroughly. Notably, the severe phenotype of impaired meiosis in PATL2 mutation group could be rescued to some extent indicated by the increased Pb1 extrusion rate (Figures 5E,F). These findings demonstrate that in oocytes, PATL2 is important for the proper translation of MOS and the activation of MAPK signaling pathway.

## Discussion

Assisted reproductive techniques (ART) have revealed previously invisible infertility phenotypes, including oocyte maturation defect. Previous studies have reported that *PATL2* mutations are responsible for oocyte maturation arrest at germinal vesicle (GV) stage and several slightly variable phenotypes (Chen et al., 2017b; Maddirevula et al., 2017; Huang et al., 2018). In this study, we identified seven different missense mutations including four novel mutations and three recurrent mutations in *PATL2* from five sporadic primary infertile cases and one family with two infertile sisters. These affected individuals had similar phenotypes but also showed a multiplicity. Although these findings make the mutational and phenotypic spectrum with PATL2 more distinct, the molecular etiology remains largely unknown. The patient (family4 II-1) was identified compound heterozygous mutations c.920G>A (p.Arg307Gln) and c.649T>A (p.Tyr217Asn), whereas an individual carried c.649T>A (p.Tyr217Asn) and c.566T>G (p.Leu189Arg) was found in previous report (Chen et al., 2017a). Although the two patients have one different mutation site in addition to p.Tyr217Asn, the phenotypes of the two are very similar including MI arrest and Pb1 oocytes with large polar body (Chen et al., 2017b). Another recurrent mutation p.Ser459Tyr is conserved among different species, but the phenotype of the patient (family 3 II-1) is not completely consistent with that of the patient in previous report (Jia et al., 2020). Considering that the PATL2^Y217N^ mutation recurred in the patients with similar phenotype and is conserved among different species, we chose p.Tyr217Asn in PATL2 (PATL2^Y217N^) as a typical to explore the possible mechanism of *PATL2* mutations that cause oocyte maturation defect.

PATL2 (P100 in Xenopus) is an RNA binding protein that contains the conserved PAT1 domain. The observation from Xenopus oocytes indicated P100 is maternal factor that expresses in growing oocytes, and declines during oocyte maturation (Nakamura et al., 2010). It was demonstrated that PATL2 level is less abundant with oocyte growth in mouse model (Christou-Kent et al., 2018). In our study, we collected oocytes at GV, MI, and MII stages, repeated the conclusion that PATL2 was highly expressed in early stages of oocytes but the abundance declined sharply, with the onset of meiosis. PATL2 conforms to the rule of maternal expression, which suggests that PATL2 has an indispensable influence on oocyte maturation and even early embryonic development. Indeed, according to previous studies in Xenopus, this decline in the level of PATL2 is necessary for meiotic maturation and that ectopic expression may impair meiosis progression (Marnef et al., 2010; Nakamura et al., 2010). While, the oocytes from affected individuals with corresponding mutations in *PATL2* showed lower level of PATL2 than did normal oocytes, and it was speculated that the reduced level of PATL2 expression was the pathogenic cause (Chen et al., 2017b; Wu et al., 2019). We doubted whether there was other possible etiology besides decreased protein level resulting from mutations. Considering PATL2 contained multiple ubiquitination sites and degraded by proteolysis (Rechsteiner and Rogers, 1996; Marnef et al., 2010), we used *in vitro* experiments to detect the mutation effect. It is worth noting that decreased ubiquitination level in the mutant groups was determined. Then, the protein level of PATL2^Y217N^ was indicated to be more stable compared to PATL2^WT^, leading to the abnormal abundance of PATL2. Next, we focused on the possible downstream influence.

In mammalian oocytes, a large amount of maternal mRNAs stored during oocyte growth, and subsequent meiotic progression required temporal maternal mRNA polyadenylation and translational activation (Piccioni et al., 2005; Chen et al., 2013). Whereas, P100, the ortholog of human *PATL2*, was described to be a partner of CPEB in Xenopus oocytes, and plays a vital role in translational repression. It was suggested that P100 represses specific translation of c-mos by observations that less protein level of MOS and fewer numbers of oocytes underwent GVBD coupled with P100 overexpression (Nakamura et al., 2010). MOS plays an extremely important role in meiotic maturation progression cause it activates MAP kinase and involves in microtubule organization in mouse oocytes, and the MOS^−/−^ oocytes underwent the first meiotic division frequently appearing symmetrical cleavage or producing an abnormally large polar body (Choi et al., 1996; Verlhac et al., 1996; Zhang et al., 2015). Coincidentally, the oocytes retrieved from patient (family4 II-1) who carried p.Tyr217Asn and the mouse oocytes injected with PATL2^Y217N^ cRNA presented a similar phenotype of anomalous polar body in MOS^−/−^ oocytes. The oocytes injected with PATL2^Y217N^ cRNA also showed partially symmetrical cleavage and abnormal spindle. Thus, we proposed that whether the abnormal abundance of PATL2 caused the decrease of MOS and the defect of oocyte maturation. Consistent with our hypothesis, the mutant PATL2 significantly affected the translation of c-Mos after injection of wild-type or mutant PATL2 cRNA indicated by western blot analysis and the 3′ UTR reporter. These observations therefore suggest that the mutation p.Tyr217Asn in PATL2 represses translation of MOS and results in decline in amount of MOS and a similar phenotype to that of MOS^−/−^ oocytes. MOS is identified as upstream activator of mitogen-activated protein kinase (MAPK), which is normally expressed throughout oocyte maturation (Ferrell et al., 1991; Pelech and Sanghera, 1992; Posada and Cooper, 1992; Shibuya et al., 1992). A MAPK cascade connects translation and degradation of maternal mRNA with meiotic progression in mouse oocytes, since ERK1/2-mediated CPEB1 phosphorylation or degradation is a main mechanism of maternal mRNA translational, and is crucial for mouse oocyte maturation (Sha et al., 2017; Chen et al., 2020). As its upstream activator was affected, we verified the impaired MAPK pathway indicated by lower pERK1/2. These results were consistent with the defect of meiotic maturation in oocytes microinjected with PATL2^Y217N^ cRNA.

In the clinic, current strategies of regular IVM to improve outcome of immature oocytes cannot exceed oocyte maturation arrest (Hourvitz et al., 2010). In addition, it was demonstrated that besides the ERK pathway recited above, other MAPK families have been identified. One of them is p38 pathway, which is activated by cellular stress (Zhang and Liu, 2002). We noticed that the phenotypes of Mos^−/−^ and Erk1/2^−/−^ oocytes are not entirely consistent, one of the possibilities is MOS might activate targets of other MAPK family members, such as p38MAPKs besides ERK1/2 (Zhang et al., 2015). However, the speculation has not yet been sufficiently explained. In our study, we tried the rational methods of IVM, adding p38 MAPK activator dehydrocorydaline chloride rather than the traditional one. The designed IVM plan greatly improved the probability of oocyte maturation *via in vitro* experiments in mouse model. The data suggest a potentially clinical application that the PATL2 mutations involved in the phenotype of oocyte maturation arrest, which need to be further assessed.

**Figure 6:**
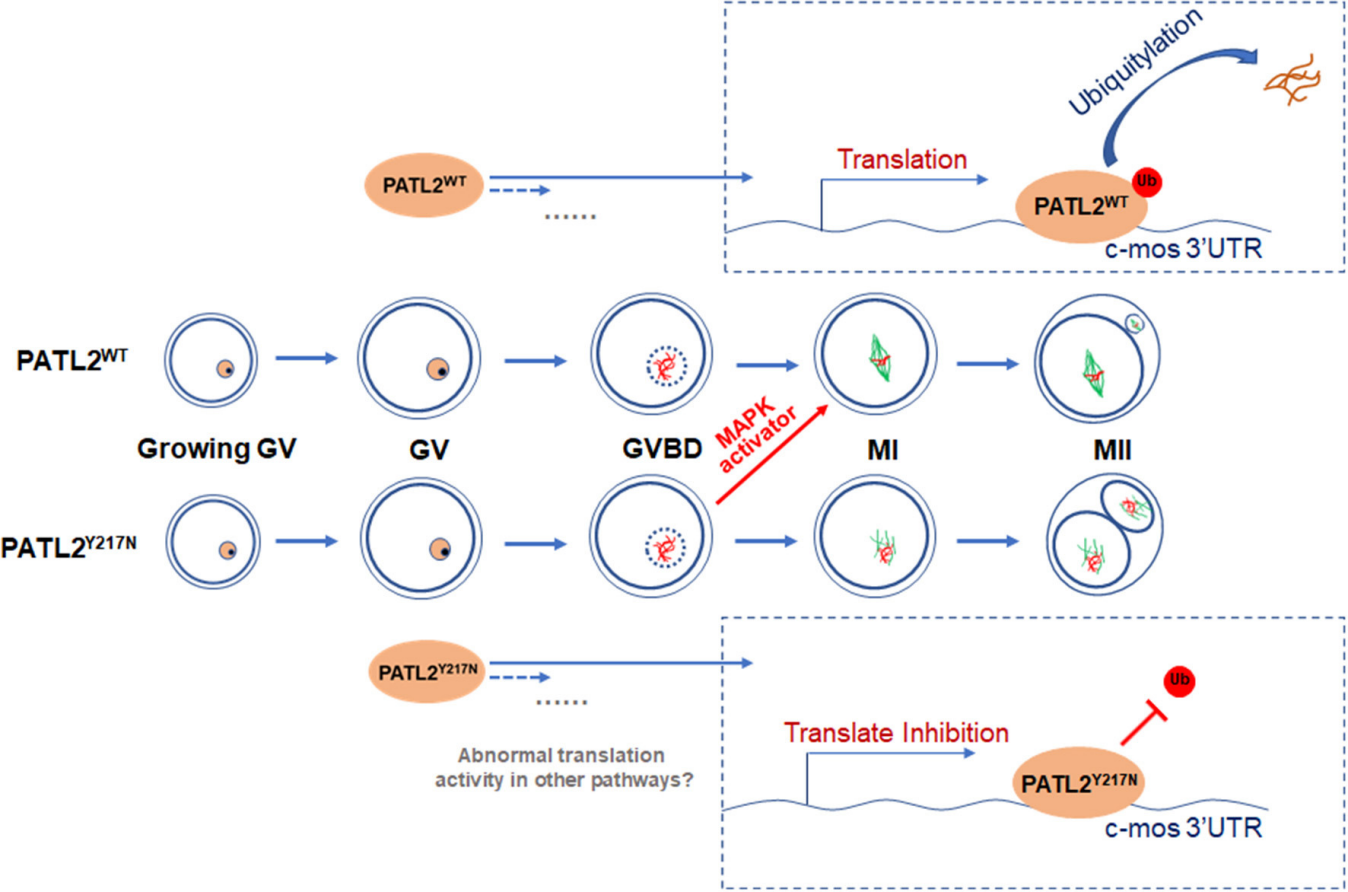
A schematic representation of the proposed mechanism by which Y217N mutation in PATL2 impairs oocyte meiotic process. The mutation p.Y217N in PATL2 decreased its ubiquitination level and led to its abnormal accumulation. Then, increased PATL2 repressed translational activity of Mos and impaired oocyte meiotic maturation. Addition of the activator of subsequent MAPK signaling partially rescued the impairment of meiotic maturation in PATL2 mutated oocytes. Further, it was speculated that some other mechanisms still remained in defect oocyte maturation influenced by PATL2 mutation.

In conclusion, our study identified several novel and recurrent mutations in PATL2 of primary infertile female and emphasized its pathogenicity and phenotypic consistency. Our observation *in vitro* contrasted with data from previous studies, where mutant PATL2 was shown rapid degradation (Chen et al., 2017b; Wu et al., 2019; Liu et al., 2020). This discrepancy suggested that the functional effects of PATL2 mutation seemed diverse. The results in this study indicate that the mutation in PATL2 decreased its ubiquitination level and led to abnormal accumulation. Then, unusually increased PATL2 repressed translational activity of MOS and impaired oocyte maturation process. As MOS does not seem to be essential for the initiation of oocyte maturation, it suggests that the oocyte maturation arrest observed in patient with PATL2^Y217N^ mutation may be caused by some other mechanisms. As PATL2 was recognized as a specific translational repressor, it might also inhibit translational activities of some pathways beside MOS/MAPK during oocyte maturation (Figure 6). Moreover, it also suggested that modified IVM protocol with p38 MAPK activator dehydrocorydaline chloride improved the maturation rate of mutated cRNA injected oocytes, which may apply for clinical use to help solve this plight in the future. Our work expands the understanding of the pathogenesis of *PATL2* gene mutations and recommends a rationally advanced IVM method in combination with PATL2 target gene diagnosis for infertile patients affected by oocyte maturation arrest.

## Data Availability Statement

The original contributions presented in the study are included in the article/Supplementary Material, further inquiries can be directed to the corresponding author/s.

## Ethics Statement

The studies involving human participants were reviewed and approved by Medical Ethics Committee of Women's Hospital of Nanjing Medical University, Nanjing Maternity and Child Health Care Hospital Ethics Committee of the First Affiliated Hospital with Nanjing Medical University, Jiangsu Province Hospital. The patients/participants provided their written informed consent to participate in this study. The animal study was reviewed and approved by Ethics Committee of Nanjing Medical University. Written informed consent was obtained from the individual(s) for the publication of any potentially identifiable images or data included in this article.

## Author Contributions

QC and CZ: conceptualization. QC: methodology and data curation. CW: software, formal analysis, and visualization. QC, CZ, and RH: validation. LC: investigation. XZ, MX, and JZ: resources. QC, CZ, and LC: writing—original draft preparation. QC, RH, and XM: writing—review and editing. QC and JH: revision. XL and XM: supervision. XL: project administration. RH, CZ, MX, and XL: funding acquisition. All authors contributed to the article and approved the submitted version.

## Conflict of Interest

The authors declare that the research was conducted in the absence of any commercial or financial relationships that could be construed as a potential conflict of interest.
