# Supplementary material for: The Recurrent Mutation in PATL2 Inhibits Its Degradation Thus Causing Female Infertility Characterized by Oocyte Maturation Defect Through Regulation of the Mos-MAPK Pathway
Source: Front Cell Dev Biol. 2021 Feb 4;9:628649. doi: 10.3389/fcell.2021.628649 (PMC7890943; doi:10.3389/fcell.2021.628649)
Supplement: Supplementary file 1 [file Table_1.docx]

Table S1: Primer sequences for sanger sequencing

| Primer name | Primer sequence |
| --- | --- |
| c.C898T (Family1) | F:GCGGTGGAGGCCTATACTTC |
|  | R:GATAGTGCTCCCACCTGCTC |
| c.A1345G (Family2) | F:GCTTCCAGGTGATCATGTGC |
|  | R:TTGACCCTCCACGAACTCCT |
| c.C1376A (Family3) | F:ACTTCACCTGGCTCAGTCTG |
|  | R:CACCAAATGCCGAGTGTGAA |
| c.A931G (Family3) | F:CTGGTTGCTTTGCTGCTCAG |
|  | R:CAGTCAGAGGCTTCGGGTATT |
| c.G920A (Family4) | F:CATCTGGGAATTTGAGGGTG |
|  | R:TGTGTTTAAGACTGTGACCG |
| c.T649A (Family4) | F:CCCCATCCTGTAGTGATTCT |
|  | R:GGTGGGTAAGTCTAAAGGGT |
| c.C1336T (Family5) | F:GCTTCCAGGTGATCATGTGC |
|  | R:TTGACCCTCCACGAACTCCT |

F forward complementation, R reverse complementation
